# Characteristic modes of a slot antenna design based on defected ground structure for 5G applications

**DOI:** 10.1038/s41598-023-42130-0

**Published:** 2023-09-15

**Authors:** Maie A. Gaber, Mostafa El-Aasser, Ashraf Yahia, Nasr Gad

**Affiliations:** 1 Physics Department, Faculty of Science, Ain Shams University, Cairo, 11566 Egypt; 2 Physics Department, Faculty of Science, Galala University, New Galala City, 43511 Egypt

**Keywords:** Engineering, Electrical and electronic engineering

## Abstract

This study employs characteristic mode analysis to investigate a defected ground antenna for multiband applications. The antenna structure incorporates three U-shaped slots in the ground plane, forming a defected ground structure. The microstrip line is exclusively present on the front side plane. The antenna is printed on a substrate made of a ceramic-filled PTFE composite with a size of 20 mm × 21 mm × 0.76 mm and a dielectric constant of 3. The proposed antenna is analyzed using the characteristic mode analysis based on the method of moment and simulated by an electromagnetic simulator based on the finite element method. A multiband antenna is fabricated and tested to validate the proposed antenna performance. The simulation and measurement results reveal that the antenna exhibits good input impedance bandwidths of S_11_ ≤  − 10 dB that extend from 2 to 12 GHz with three bands around the operating frequencies (2.96, 6.06, and 8.03) GHz.

## Introduction

Antennas are crucial to the fast development of wireless communication systems. Multiband printed antennas have been the research priority with this development. Printed antennas have the advantages of low profile, low cost, lightweight features, easy manufacture, and integration with microwave monolithic integrated circuits (MMICs)^1,2^.

In the past decades, various techniques have been widely employed to design multiband printed antennas based on the defected microstrip structure (DMS), defected ground structure (DGS), meander lines, and notches. These techniques are employed to improve the performance parameters of conventional microstrip/printed antennas for multiband applications. The DGS has been the most widely employed technique in the last two decades. It is implemented by making a defect/etching on the ground plane of microstrip circuits. The defect of DGS can be non/periodic elements or a unit cell^3,4^. The usage of DMS and DGS for realizing the design of a mirror stairs microstrip multiband antenna that covers the range of 2–17 GHz is explained in^5^. A printed slot antenna with defects in the ground plane that produce five bands was successfully designed in^6^. Different Euclidean slot forms printed on the ground plane with and without Euclidean patches were used to investigate printed slot antennas^7^. Two rectangular slots defected on the ground plane and five strips located inside the two slots were employed to operate at four bands (2.44–2.79, 5.88–6.39, 7.5–8.25, 11.27–12.69) GHz in^8^.

Characteristic mode analysis (CMA) was recently developed from the characteristic mode theory (CMT). CMA allows a physical insight into the radiation emitted by a multiband antenna and demonstrates how to design an efficient radiating topology that offers a multiband characteristic. The CMT depends on the electric field integral equation based on the method of moment (MoM) formulation^9^. CMA was applied to design an antenna at 2.4 GHz based on a single meander line in the ground as DGS^10^. A compact and efficient broadband antenna with two rectangular slots and a reduced ground plane is presented in ^11^. The performance of this antenna is analyzed using CMT for various radio systems. This paper^12^ presents a simple compact circularly polarized slot antenna with a wide bandwidth for wireless applications. Its performance is demonstrated using CMA as well as some experimental measurements. A design of a filtering antenna for 5G FR2 band based on CMA of the radiation and feeding structures is presented. Its performance is validated by fabrication and measurements in^13^. In the study of^14^, CMT defined the feeding structures and positions for two radiating ground antennas containing simple feeding loops. In^15^, the CMT was employed to design a compact and wideband circularly polarized antenna predicting the feeding position. CMA was employed to improve the performance of smartwatch antennas^16^.

In this work, we present a novel and compact multiband antenna that effectively covers three specific frequency bands relevant to 5G and ITU applications. Our approach incorporates CMA and DGS techniques to facilitate the design and optimization of the antenna. The proposed antenna consists of a defected copper plane with a rectangular slot and three U-shaped slots of different dimensions, creating a DGS that controls the current distributions and the modal behavior of the antenna. The CMA, based on the MoM, is applied to design and optimize the antenna’s characteristics using the equations of CMT. The antenna is simulated using a finite element method (FEM) based electromagnetic simulator and provides three bands with resonance frequencies at 2.96 GHz, 6.06 GHz, and 8.03 GHz. The first and second bands are in the region of 5G sub-7GHz: (2.89–3.03) GHz, and (5.99–6.14) GHz. The third band (7.84–8.21) GHz is appropriate for ITU-8 GHz band communication services. This work demonstrates a novel methodology for multiband antenna design with physical insight, without a substrate, that stands in contrast to conventional antenna design methods that rely on trial-and-error approaches without a comprehensive understanding of the underlying physics. In the subsequent section, we present the key equations derived from CMT that hold particular significance. The proposed antenna undergoes examination using two distinct techniques: firstly, the CMA is employed to achieve the final design of the antenna, following a comprehensive five-step process guided by the equations of CMT. In the subsequent part, the FEM technique is utilized to conduct additional parametric investigations on the proposed antenna. Experimental results are then discussed to validate the simulated outcomes obtained from both the CMA and FEM methods. Finally, a dedicated conclusion section summarizes the findings and implications of the study.

### Characteristic mode theory

The CMT was first proposed by Robert Garbacz^17^ in 1971 and then investigated by Roger Harington^18^. It provides the response in terms of eigenvalues, surface current, field radiation, characteristic angle, and modal significance to search for dominant resonating modes. These modes are excitation independent and dimensions dependent. The following eigenvalue equation is used to estimate the characteristic modes of a conducting surface.

1 $$XJ_{n} = \lambda_{n} RJ_{n}$$

where J_n_ is the eigencurrent, λ_n_ is the eigenvalue of the nth characteristic mode. The complex impedance Z is defined as Z = R + jX, such that R and X are the Hermitian real and imaginary components of the matrix Z, respectively. The characteristic angle (α_n_) represents the phase difference between J_n_ and the electric field E_n_ and can be calculated by

2 $$\alpha_{{\text{n}}} = {18}0^{^\circ } - {\text{arctan}}\left( {\lambda_{{\text{n}}} } \right)$$

Resonance modes exist when λ_n_ = 0, therefore α_n_ must be equal to 180°; otherwise, the modes are associated with stored energy. Modal significance (MS) presents the normalized current amplitude with a range from 0 to 1 that can be calculated as

3 $${\text{MS}} = \left| {\frac{1}{{1 + \lambda_{n} }}} \right|$$

At resonance mode (λ_n_ = 0), the MS should be equal to 1.

### Analysis of the proposed antenna

**Figure 1:**
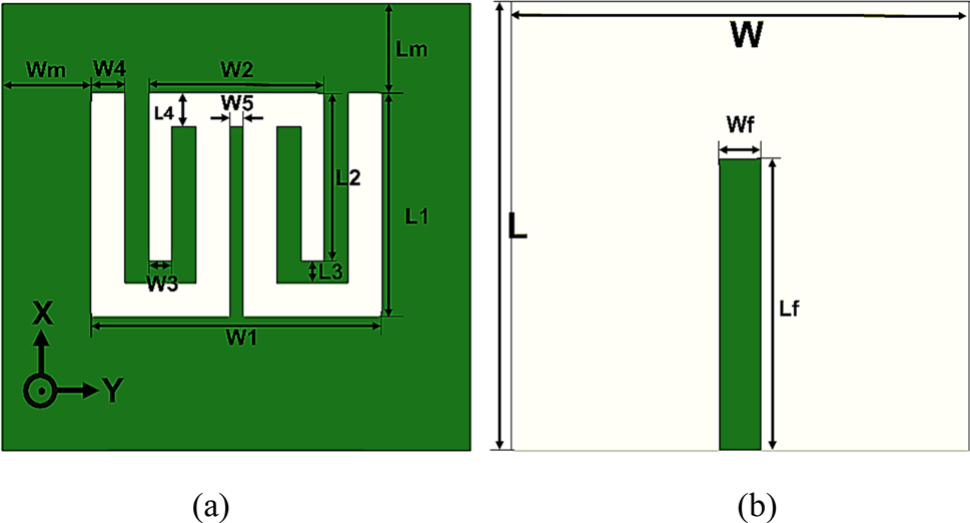
The multiband printed antenna. (**a**) Back view, and (**b**) front view.

The proposed printed multiband antenna’s geometry is demonstrated in Fig. 1. The proposed antenna has a RO3003 substrate with a size of (21 × 20 × 0.76) mm^3^ which corresponds to (0.32 λ_g_ × 0.30 λ_g_ × 0.01 λ_g_), a dielectric constant (ε_r_ = 3), and tangent loss (tan δ = 0.0013). At the top of the substrate, there is only a microstrip line of feeding length (Lf) and width (Wf). Moreover, at the substrate’s bottom, there is a ground plane of the proposed antenna that involves three U-shaped defected slots. The slots are located in a rectangular shape etched with dimensions (13 × 10) mm^2^. There is a middle vertical strip with a length equal to 8.5 mm and a width (W5 = 0.6 mm), which is under and parallel to a transmission line. The narrow strip evenly divides the slots that consist of two identical U-shaped slots with length (L1 = 10 mm) and width equal to 6.2 mm. With (W4 = 1.5 mm), the arm slot width for the right and left U-shaped slots are kept fixed. The length (L2 = 7.5 mm) and the width (W2 = 7.8 mm) of the inverted third U-shaped are etched. The vertical arms of the inverted U-shaped slot have a width (W3 = 1 mm). All parameters of the proposed printed antenna are shown in Table 1.

**Table 1:** Optimized parameters of the proposed antenna (in mm).

L	W	Lf	Wf	Lm	Wm	L1	W1
20	21	13	1.92	4	4	10	13

L2	W2	L3	W3	L4	W4	W5	
7.5	7.8	1	1	1.5	1.5	0.6	

The slot antenna is commonly employed for wideband and multiband applications aside from designing the printed slot antenna’s slot length, the radiating element must be around λ_g_/2 to realize a multiband operation for the printed antenna ^19^.

The guide wavelength (λ_g_) is given by:

4 $$\lambda_{g} = \frac{c}{{f\sqrt {\varepsilon_{reff} } }}$$

5 $$\varepsilon_{reff} \cong \frac{{\varepsilon_{r} + 1}}{2}$$

where ε_reff_ is the effective dielectric constant.

#### CMA for the design procedure

The CMA is applied to the antennas for understanding the EM mode behavior of the design. In this analysis, the ground plane of the antennas is merely considered, disregarding the substrate and transmission line.

**Figure 2:**
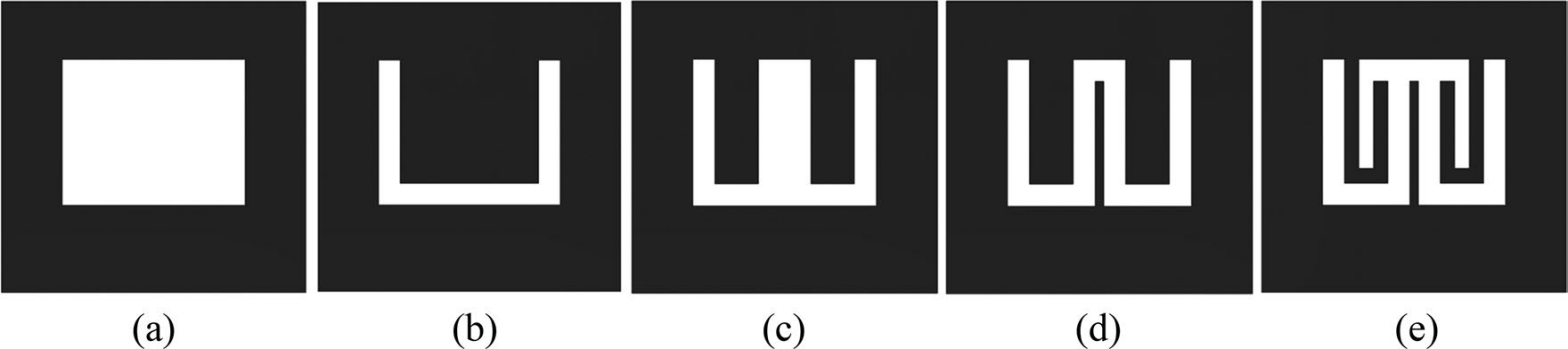
Design steps of the proposed antenna. (**a**) Antenna 1, (**b**) Antenna 2, (**c**) Antenna 3, (**d**) Antenna 4, and (**e**) Antenna 5.

Since there is no substrate material in CMA, the dielectric constant, ε_r_ = 1 (air). In processing the proposed multiband antenna design, five prototypes are considered as demonstrated in Fig. 2. In Antenna 1, Fig. 2a, a primary rectangular slot is cut on the ground plane with the dimensions (13 × 10) mm^2^. A rectangular patch is placed in the primary rectangular slot for Antenna 1 with dimensions (10 × 8.5) mm^2^ to create a U-shaped slot in Antenna 2 as shown in Fig. 2b. In the center of a U-shaped slot, a rectangular slot is etched with size (3.6 × 8.5) mm^2^, and an E-shaped slot is formed as Antenna 3 as in Fig. 2c. A narrow strip is added to the center arm of the E-shaped slot, and double U-shaped connected slots are made to create Antenna 4 in Fig. 2d. Finally, in Fig. 2e, an inverted U-shaped slot is added to create Antenna 5 (the proposed antenna).

**Figure 3:**
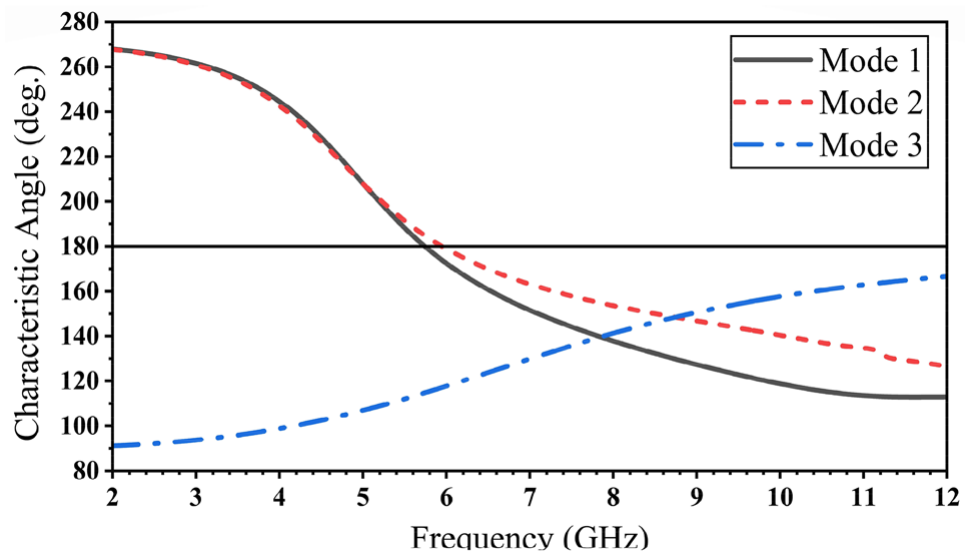
The characteristic angles of the first three modes for Antenna 1.

**Figure 4:**
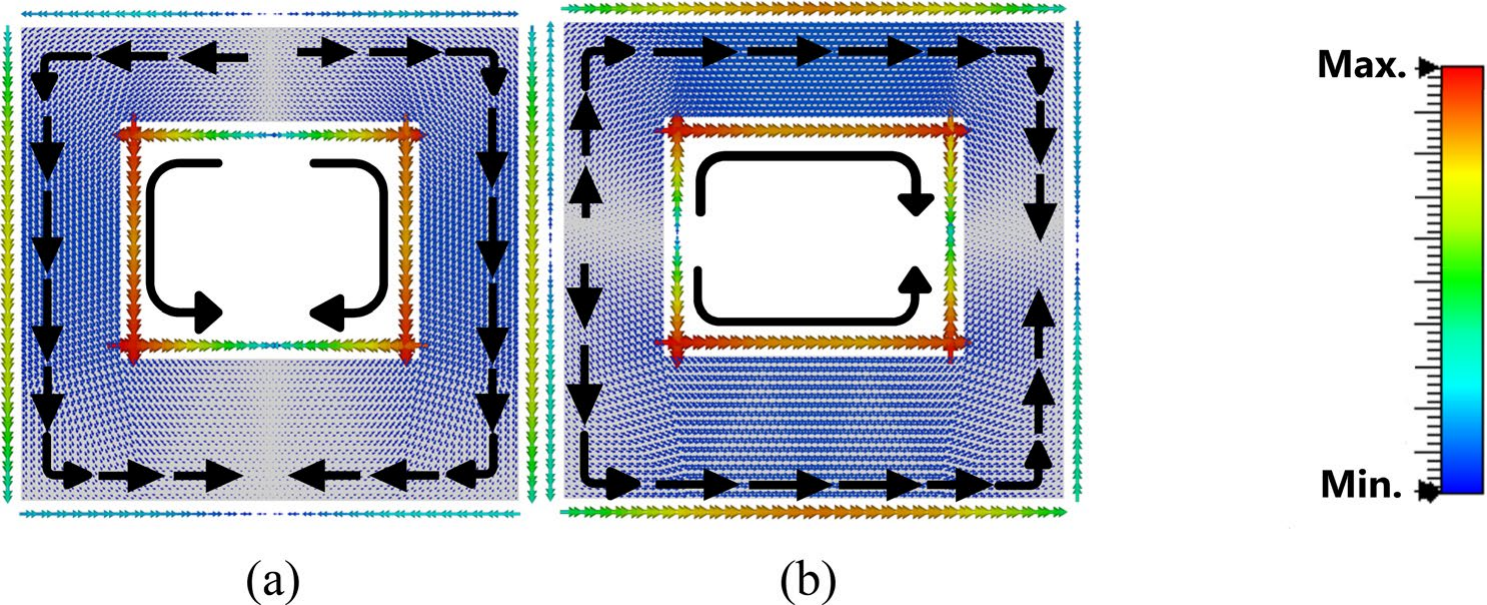
The current distribution for Antenna 1. (**a**) Mode 1 @ 5.74 GHz, (**b**) Mode 2 @ 5.95 GHz.

**Figure 5:**
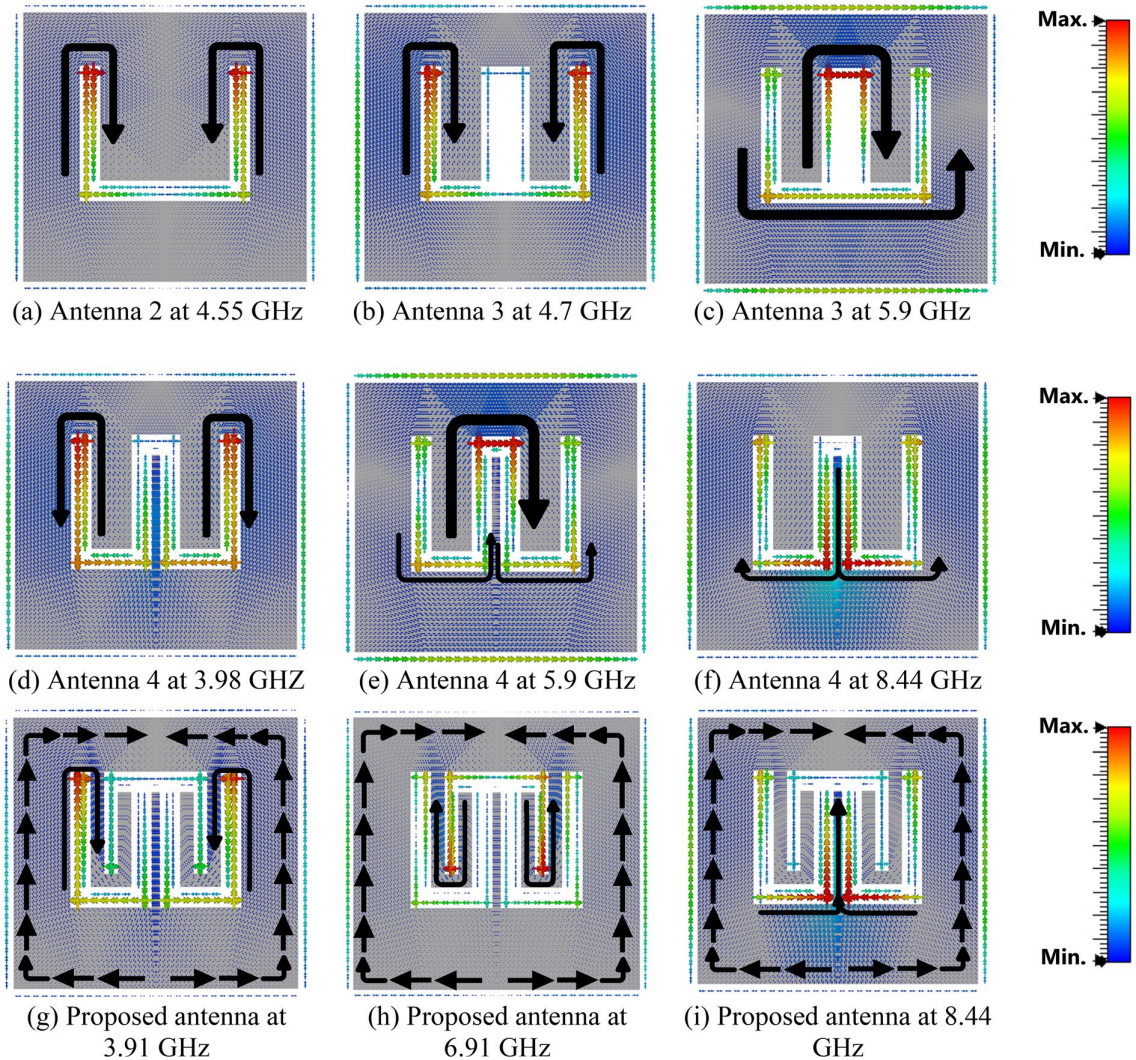
The current distribution for the characteristic modes in antenna procedure.

The first three modes are investigated using software based on MoM to solve the characteristic modes at a specific frequency. Figure 3 demonstrates the characteristic angle versus frequency. With the condition of a resonance mode when λ_n_ = 0, α_n_ must be equal to 180° indicating that the first two modes are at (5.74 and 5.95) GHz. The existing mode will be according to the transmission line’s position. The eigencurrents’ profiles for Antenna 1 are an explanation for how to design the feeding line (vertical or horizontal), to excite the first or the second mode (5.74, 5.95) GHz as demonstrated in Fig. 4a,b, respectively. According to the characteristic angle, the current distributions are obtained for each antenna as demonstrated in Fig. 5. The dominant mode in antenna 2 is established at 4.55 GHz, as demonstrated in Fig. 5a. In Antenna 3, the mode at 4.7 GHz is created due to the high current concentration at the left and right arms of the E-shape, while the mode 5.9 GHz is resonating due to the high current concentration at the middle arm of the E-shape, as depicted in Fig. 5b,c, respectively. Adding a narrow strip in antenna 4 causes some changes in the resonating frequencies, Fig. 5d,f, while the second frequency at 5.9 GHz remains the same, Fig. 5e. The 4.7 GHz of Antenna 3 is shifted to 3.98 GHz as the current path is elongated, as in Fig. 5d. The frequency at 8.44 GHz is created due to the high current concentration around the added strip, as in Fig. 5f. The etched U-shaped to form the final design (Antenna 5) created the second resonance frequency at 6.91 GHz, as demonstrated in Fig. 5h while the first and second resonance frequencies remain almost the same at (3.91, 8.44) GHz, respectively.

**Figure 6:**
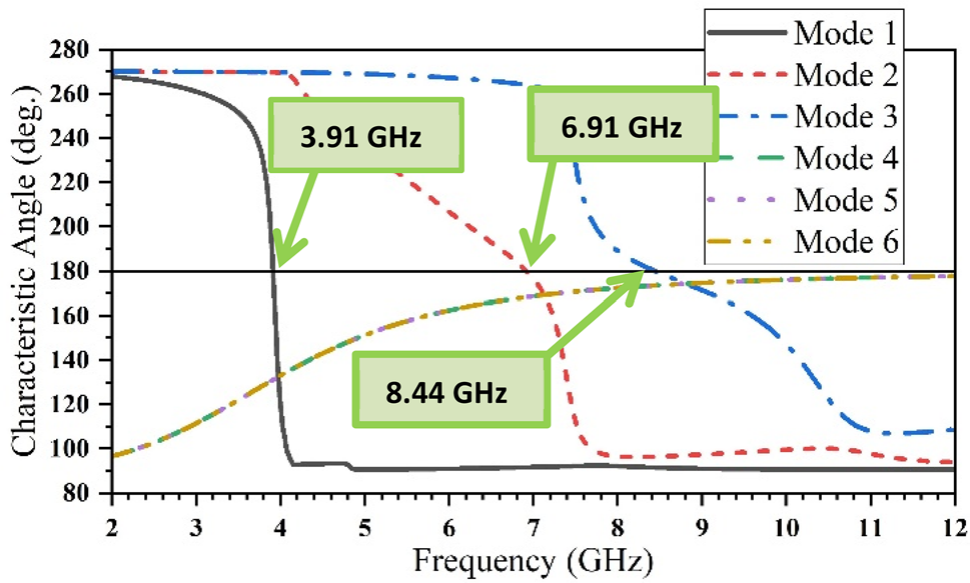
The characteristic angle for the six resonance modes of the proposed antenna.

The characteristic angles for the first six modes are shown in Fig. 6. The resonant modes at its eigenangle crosses 180° are (3.91, 6.91, and 8.44) GHz. The characteristic angle condition is not obeyed by the other modes.

**Figure 7:**
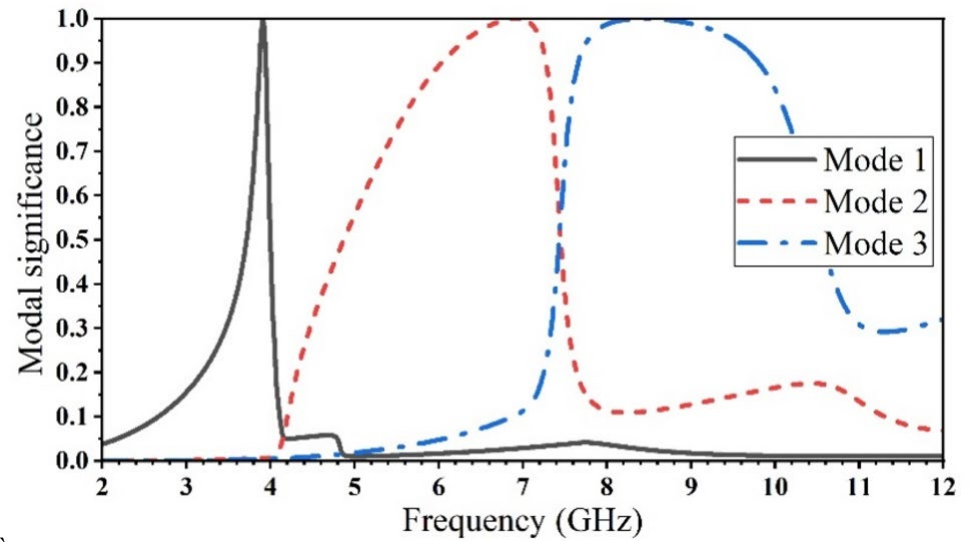
The modal significance for the three resonance modes of the proposed antenna.

The MS is calculated for the three resonance modes by using equation (3), as demonstrated in Fig. 7, in which three various resonating areas coexist, at (3.91, 6.91, and 8.44) GHz. According to the proposed antenna structure, the resonance areas have relative impacts. The inverted U-shaped slot (resonating at 6.91 GHz) area and the area between the internal arms of U-shaped slots (resonating at 8.44 GHz) have the most significant effect on each other.

#### Parametric study

**Figure 8:**
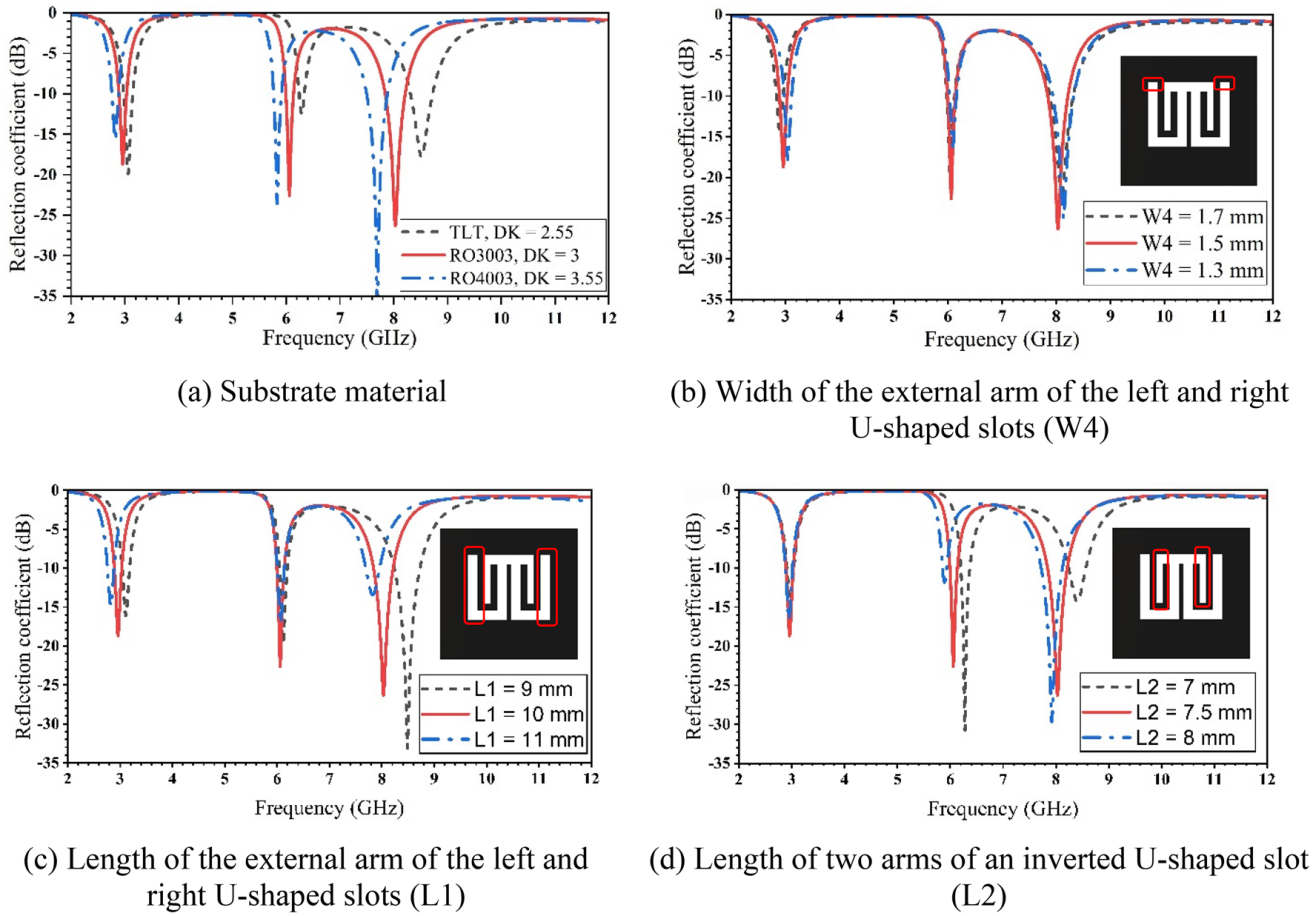
The simulated reflection coefficient for the proposed antenna with various configurations.

A high-frequency structure simulator based on the FEM was employed to optimize the antenna’s performance^20^. Figure 8 demonstrates the reflection coefficient of the proposed antenna for various parameters. Three different substrate materials are investigated to demonstrate the effect on the operating frequencies, as indicated in Fig. 8a, which confirms the inverse relationship between the dielectric constant and the resonant frequency as given by equation (4). It shows a decrease in frequencies as the dielectric materials increase. Figure 8b illustrates the effect of changing (W4) by increasing or decreasing 0.2 mm from the width of the proposed antenna (W4 = 1.5 mm). In Fig. 8c, the first and third frequencies are shifted when the length (L1) is changed by 1 mm to give L1 = 9, 10, and 11 mm. This can be explained by the change in the current distributions along the antenna arms as shown in Fig. 5. The length L2 (= 7.5 mm) is increased and decreased by 0.5 mm, leading to a shift in the second and third resonance frequencies, as seen in Fig. 8d.

## Results and discussion

**Figure 9:**
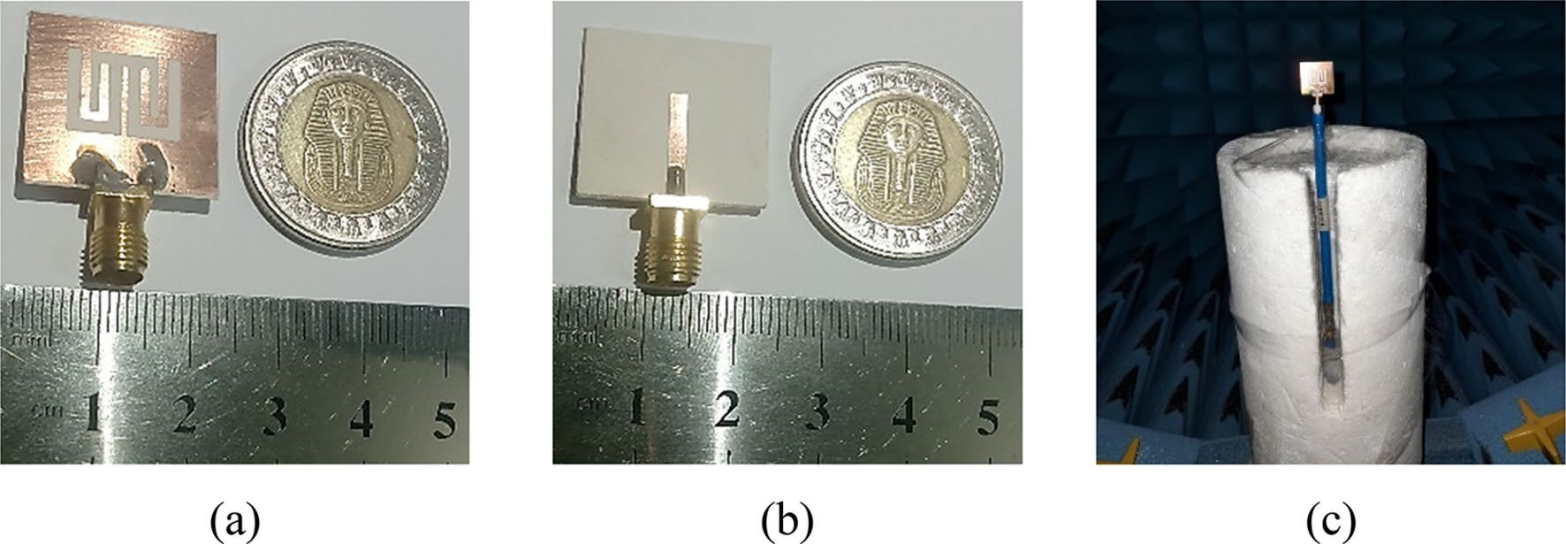
The fabricated antenna. (**a**) Back view, (**b**) front view, and (**c**) the antenna in the measurement setup.

A novel proposed antenna was designed, fabricated, and measured. The electrical performance parameter (S_11_) was measured by using a Rohde & Schwarz ZVB 20 Vector Network Analyzer. The top and bottom sides of the fabricated antenna are illustrated in Fig. 9a,b. The antenna is shown inside the anechoic chamber in Fig. 9c.

**Figure 10:**
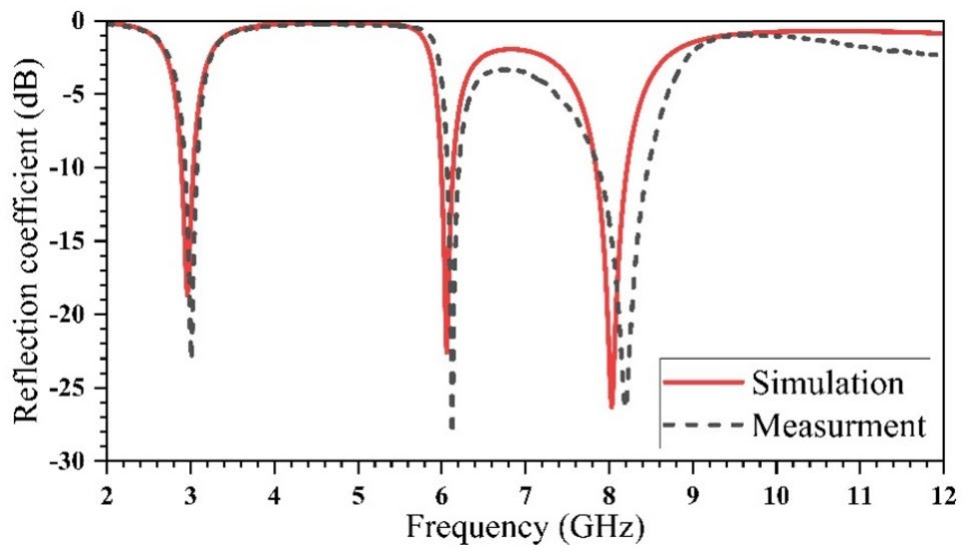
Return loss of simulated and measured proposed antenna.

The simulated and measured results of the reflection coefficient (S_11_) are shown in Fig. 10. There is a good agreement between the simulation and measurement results in the region from 2 to 12 GHz. The measured impedance bandwidths are 2.93–3.07 GHz with a bandwidth of 0.14 GHz (4.6%), 6.07–6.21 GHz with a bandwidth of 0.14 GHz (2.28%), and 7.87–8.47 GHz with a bandwidth of 0.6 GHz (7.23%) for |S_11_|≤ − 10 dB.

**Figure 11:**
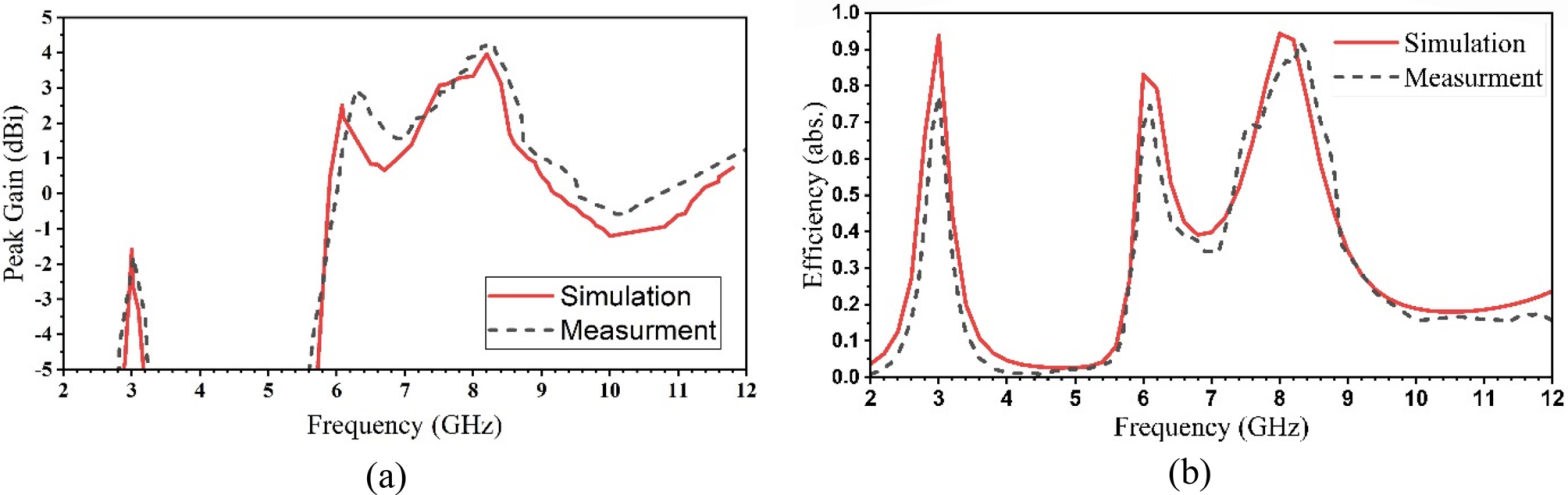
The simulated and measured results of (**a**) peak gain and (**b**) radiation efficiency.

The proposed printed antenna’s peak gains and normalized efficiencies are simulated and measured, as shown in Fig. 11a,b, respectively. Table 2 presents the gain and efficiency results at each frequency.

**Table 2:** The simulated and measured results of peak gain and efficiency.

	Simulated			Measured		
Frequency (GHz)	2.96	6.06	8.1	3	6.12	8.2
Gain (dBi)	− 1.5	2.51	3.96	− 1.7	2.9	4.2
Efficiency (abs.)	0.93	0.83	0.94	0.77	0.81	0.92

**Figure 12:**
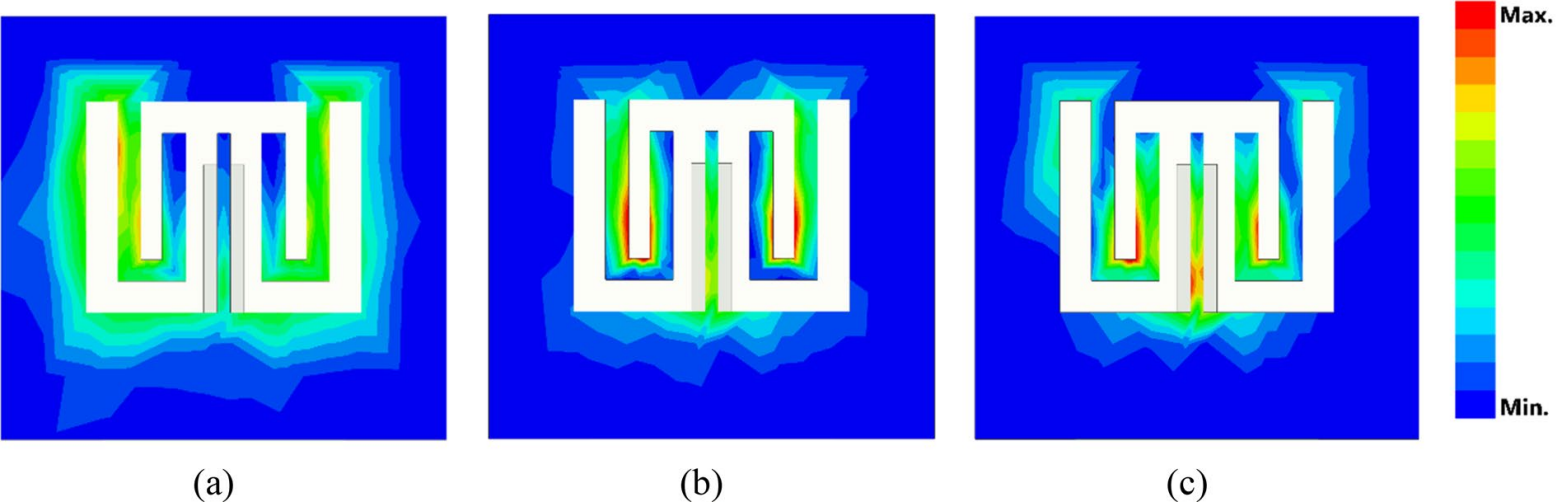
The current distribution over the proposed multiband printed antenna at (**a**) 2.96 GHz, (**b**) 6.06 GHz, and (**c**) 8.03 GHz.

Figure 12 shows the current distribution for the simulated antenna, considering the existence of the microstrip line and dielectric material (ε_r_ = 3), at resonance frequencies (2.96, 6.06, and 8.03) GHz. The resonating areas obtained in Fig. 12 are the same as those in Fig. 5g–i, when the microstrip line and substrate material were absent.

The proposed antenna is compared with published multiband antennas and summarized in Table 3. Noting that all these multiband antennas were not designed with CMA. The held comparison shows that the proposed antenna is compact compared to the others.

**Table 3:** A comparison of the proposed work with the state-of-the-art published works.

References	Size (mm × mm × mm)	Resonance frequencies (GHz)	gain (dbi)	Substrate	Design method
^21^	27.5 × 20 × 1.5	2.44, 3.55, 5.6	3.9, 4.1, 3.8	FR4	DMS, monopole
^22^	33 × 17 × 1.6	2.5, 3.5, 5.5	2, 2, 3.3	FR4	Ring monopole, slots
^23^	57.2 × 31.2 × 1.6	0.8, 2.45, 3.5, 5.5	− 8.12, − 1.31, 1.46, 3.66	FR4	E-CRLH unit cell, CPW
^24^	43 × 33 × 1.6	2.45, 2.8, 3.8, 5.5	5.5, 4.4, 0.0, 5.6	FR4	DGS
^25^	32 × 15 × 1.6	1.8, 2.4, 3.35, 5.4	1.5, 1.7, 2.5, 3	FR4	DMs, DGS, monopole
Prop	20 × 21 × 0.76	3, 6.1, 8.2	− 1.5, 2.51, 3.96	RO3003	DGS

**Figure 13:**
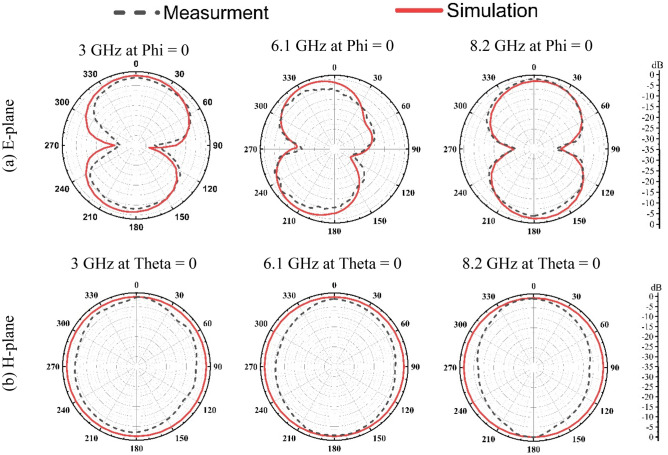
The simulated and measured radiation patterns of the proposed multiband printed antenna.

The simulated and measured radiation patterns of the proposed antenna for an E-plane (Y–Z plane) and H-plane (X–Z plane) are shown in Fig. 13. A StarLab System was used to measure E-field and H-field patterns.

## Conclusion

A printed antenna was specifically designed to cater to 5G multiband applications. The antenna design incorporates three U-shaped slots strategically placed as defects within the ground plane, while the transmission line is implemented on the top of the substrate. The performance of the designed antenna is analyzed and simulated through the utilization of CMA based on the method of moments and FEM (finite element method) simulators. The fabricated antenna was constructed on a RO3003 substrate, featuring dimensions of (21 × 20) mm^2^. This fabrication process served as a means to validate the accuracy and reliability of the simulation results. The experimental evaluation of the fabricated antenna revealed its operational capabilities across three distinct frequency bands. Specifically, the antenna operated within the frequency ranges of (2.93–3.07) GHz with a bandwidth of 0.14 GHz, (6.07–6.21) GHz with a bandwidth of 0.14 GHz, and (7.87–8.47) GHz with a bandwidth of 0.6 GHz. The measured results obtained from the experiments demonstrated a favorable agreement with the simulated outcomes. The successful alignment between the measured and simulated results signifies the suitability of the printed antenna for communication service applications within the 5G Sub-7 GHz and ITU-8 GHz bands. These findings highlight the potential practical applications of the designed antenna in meeting the communication demands of these specific frequency ranges.

## Data Availability

The datasets generated and/or analyzed during the current study are available from the corresponding author upon reasonable request.
